# Heart Transplantation in Nonoperable Multiple Giant Coronary Artery Aneurysms: A Case Report

**DOI:** 10.1155/cric/9933683

**Published:** 2026-07-29

**Authors:** Raffaele Abete, Roberta Simona Cattaneo, Attilio Iacovoni, Claudia Vittori, Roberta Sebastiani, Ottavio Zucchetti, Francesco Moretti, Giuseppe Muscogiuri, Sandro Sironi, Amedeo Terzi, Michele Senni

**Affiliations:** ^1^ Department of Transplant Surgery and Surgical Treatment of Heart Failure, Azienda Socio Sanitaria Territoriale Papa Giovanni XXIII, Bergamo, Italy; ^2^ Department of Cardiology, Azienda Socio Sanitaria Territoriale Papa Giovanni XXIII, Bergamo, Italy; ^3^ Division of Invasive Cardiology, Azienda Socio Sanitaria Territoriale Papa Giovanni XXIII, Bergamo, Italy; ^4^ Department of Radiology, Azienda Socio Sanitaria Territoriale Papa Giovanni XXIII, Bergamo, Italy

**Keywords:** cardiac computed tomography angiography, coronary artery aneurysm, heart transplant, ischemic cardiomyopathy

## Abstract

Coronary artery aneurysm (CAA) is an uncommon dilation of a coronary artery, with a prevalence of 0.3%–4.9% in patients undergoing coronary angiography. Giant CAAs, defined as dilations greater than four times the size of normal adjacent vessels or a diameter > 8 mm, are even rarer. We report a case of a 62‐year‐old man with chronic ischemic heart disease presenting with multiple giant CAAs. The patient had a history of myocardial infarction, obesity, dyslipidemia, and was an ex‐smoker. Coronary angiography and cCTA revealed giant aneurysms in the right coronary and circumflex arteries, with extensive endoluminal thrombosis and compression of adjacent structures. The coronary anatomy was deemed unsuitable for surgical or percutaneous intervention; thus, the patient was listed and underwent orthotopic heart transplantation, without significant postoperative complications. This case highlights the complexity of giant CAAs management in adults, where conventional interventions are not feasible. The successful heart transplantation and favorable postoperative outcomes underscore the potential viability of this treatment. This case contributes to the limited literature on heart transplantation as a therapeutic option for nonoperable CAAs.

**Learning Objectives**

1. To acknowledge the potential role of cardiac transplant as a therapeutic alternative for nonoperable CAAs
2. To underline the potential benefits of pre‐procedural multimodality imaging to better clarify complex anatomy and surgical planning.

## 1. Introduction

Coronary artery aneurysm (CAA) is a localized irreversible dilation of a coronary artery, that is, at least 1.5 times larger than the adjacent normal segment. CAAs are an uncommon finding, with a prevalence of 0.3%–4.9% of patients undergoing coronary angiography and 1.4% in postmortem examinations, with a slight (40.4%) predilection for the right coronary artery [1]. Even rarer is the occurrence of a giant CAA, defined as a dilation greater than four times the size of normal adjacent vessels, or a diameter > 8 mm [2].

## 2. Case Report

### 2.1. History of Presentation

In this report, we describe the case of a 62‐year‐old man with chronic ischemic heart disease who was admitted to our cardiac surgery department in February 2024 for investigations for multiple giant CAAs. The patient was obese, had dyslipidemia, was an ex‐smoker, and had no significant comorbidities except for a left inguinal hernia that was repaired with hernioplasty in 2019. He had no known history of Kawasaki disease (KD) in childhood or other acknowledged conditions associated with the development of coronary aneurysms. However, given the extreme dimensions of the aneurysms and the early onset of symptomatic coronary artery disease, a previously unrecognized or subclinical KD remains a highly plausible etiology. He was on home therapy with bisoprolol, rosuvastatin, evolocumab, and warfarin.

### 2.2. Past Medical History

The patient was affected by chronic ischemic heart disease resulting from a previous myocardial infarction in 1991. After discharge, the patient underwent regular annual follow‐up until February 1998 when, due to the onset of exertional chest pain, he underwent a myocardial scintigraphy, which documented reversible ischemia in the infero‐posterior, basal anterior, and midbasal anterolateral regions. Consequently, a new coronary angiography was performed, revealing severe and diffuse coronary disease characterized mainly by significant and irregular dilation of the coronary vessels, with slowed intravascular flow. Transthoracic echocardiography (TTE) showed preserved biventricular function in the absence of significant valvular defects. Considering the clinical situation and the complex coronary anatomy, it was decided to optimize medical therapy by adding an oral anticoagulant to the treatment regimen. The patient subsequently remained asymptomatic with stable clinical conditions since June 2023, when he reported worsening exertional dyspnea (NYHA Class IIb). A follow‐up coronary computed tomographic angiography (cCTA) scan in May 2023 showed progression of the known severe aneurysmatic coronary artery disease, complicated by intravascular thrombosis and determining compression on adjacent structures. A cardiac magnetic resonance (CMR) without contrast confirmed the presence of preserved biventricular function, with extrinsic compression of the heart chambers by the aneurysmal coronary arteries. The patient was subsequently referred for an urgent surgical consultation and was then admitted to our cardiac surgery department for further investigations to define the best therapeutic planning.

### 2.3. Investigations

**Figure 1:**
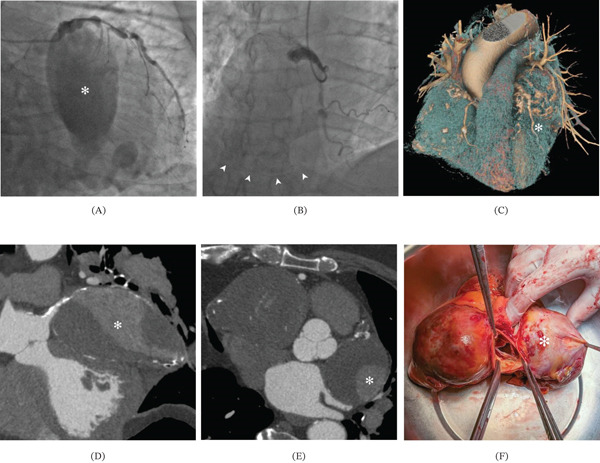
Multimodality imaging and anatomical specimen of giant aneurysms of left circumflex and right coronary arteries. (A) ICA caudal view showing a GCA of the LCX and the anterior descending artery with multiple smaller aneurismatic tracts interrupted by subocclusive stenosis. (B) 30° right anterior oblique view of a GCA of RCA with arrowheads marking its inferior border. (C) 3D reconstruction from CCTA of both aneurysms. (D) CCTA double oblique view of the LCX aneurysm. (E) CCTA axial view of both aneurysms corresponding to the anatomical specimen in panel (F). Asterisks (✻) mark the LCX aneurysm across all panels. Abbreviations to be reported: CCTA, coronary computed tomography angiography; GCA, giant coronary aneurism; ICA, invasive coronary angiography; LCX, left circumflex artery; RCA, right coronary artery.

He underwent coronary angiography, which showed giant aneurysms of the right coronary artery (occluded) and the circumflex artery, as well as proximal left anterior descending artery subocclusion. (Figure 1A,B).

We also repeated a cCTA, which confirmed the presence of multiple severe CAAs (Figure 1C–E):

- A large aneurysm of the right coronary artery in the mid and distal segments, with maximum diameters of approximately 77 × 71 mm, with nearly complete thrombotic apposition, without evidence of canalization in the delayed scans, determining compression on the right atrium and indenting the aortic bulb.
- Severe calcific atherosclerosis of the proximal circumflex artery followed by severe aneurysmal dilation in the mid segment with maximum transverse diameters of approximately 59 × 61 mm, characterized by significant thrombotic apposition with a patent lumen and maximum diameters of 30 × 35 mm, causing a compressive effect on the left atrium, left superior pulmonary vein, coronary sinus, and indenting the aortic bulb.
- Chronic calcific occlusion of the anterior descending artery in the mid segment.

The coronary anatomy was deemed extremely complex and thus unsuitable for surgical correction via coronary artery bypass grafting (CABG), as there were no viable distal branches in any of the vessels affected by the disease. The extreme size of the aneurysms and the presence of extensive endoluminal thrombosis also precluded percutaneous intervention. After a multidisciplinary discussion ruling out the possibility of intervening through both conventional percutaneous and cardiac surgical approaches, we decided to consider the patient for orthotopic heart transplantation, pending the execution of screening tests.

Cardiac catheterization showed a baseline hemodynamic profile characterized by normal cardiac index and normal left ventricular filling pressures. Pulmonary circulation pressures and resistances, right atrial pressure, and systemic resistances were also normal.

### 2.4. Surgical Intervention

On June 25, 2024, the patient underwent orthotopic heart transplantation using the atrial cuff anastomosis technique. At the level of the right coronary, left circumflex, and left anterior descending arteries, there is evidence of arterial ectasia accompanied by thinning of the tunica media and a moderate‐to‐severe increase in intraparietal and perivascular inflammatory infiltrate (Figure 1F).

## 3. Discussion

In the adult population, atherosclerotic disease is a cause of CAAs, whereas KD is responsible for most cases in children [1]; however, the morphological characteristics in this case—specifically the development of “giant” aneurysms measuring up to 77 × 71 mm—are extraordinarily atypical for purely atherosclerotic degeneration. Other causes include congenital heart disease, trauma, collagenopathies, Takayasu arteritis, polyarteritis nodosa, syphilitic aortitis, scleroderma, systemic lupus erythematosus, Behçet disease, and fibromuscular dysplasia [3]. In several case reports, CAAs have also been reported as a sequel of percutaneous coronary interventions [2]. Although the patient presented with traditional cardiovascular risk factors, these are unlikely to explain the extreme aneurysmal phenotype. The patient′s history of myocardial infarction in 1991 at a relatively young age, combined with the diffuse coronary dilatation and slowed flow documented in 1998, strongly supports the hypothesis that this case represents the late‐stage sequelae of unrecognized childhood KD. Notably, a lack of documented childhood history does not exclude KD, as many adult patients were never diagnosed during the era preceding standardized diagnostic criteria.

The pathophysiologic mechanism of CAA development remains controversial, but it likely resembles that of larger vessel aneurysms: destruction of the arterial media, thinning of the arterial wall, increased wall stress and progressive dilation of the coronary artery segment. Stenotic coronary atherosclerosis and CAAs may commonly coexist, as arteriosclerotic modifications reduce the vessel′s resistance to intraluminal pressure predisposing it to dilation [4].

As in this case, most patients with CAAs are asymptomatic unless they have significant concomitant atherosclerotic coronary artery disease or develop complications. The most observed complications are thrombosis, distal embolization, aneurysm rupture, coronary spasm, and compression of adjacent structures [5]. Consequently, the most frequent clinical presentation of patients with symptomatic CAAs is chest pain with features of stable angina, but patients may also present with myocardial infarction (both ST and non‐ST elevation), congestive heart failure, sudden cardiac death, cardiac tamponade secondary to aneurysm rupture or, in case of giant CAAs, with symptoms related to the compression of adjacent structures [6].

Coronary angiography is the gold standard for diagnosing CAAs; however, it is an invasive exam with associated risks, and the true size of the coronary aneurysms may be underestimated in case of thrombosis, as it happened in our patient. Additional information may be provided by cCTA or CMR, noninvasive techniques that allow precise definition of the size, shape, and location of the aneurysm, and the three‐dimensional relationships with adjacent anatomical structures [4, 7]. CMR can also be used to assess biventricular size and function, evaluate myocardial perfusion, and identify the presence of myocardial scarring [8]. An approach based on multimodality imaging was hence essential for surgical planning.

To date, there are no randomized clinical trials and therefore no standardized recommendations for the management of CAAs, as most of the available data in literature are derived from case series and anecdotal evidence. Multiple therapeutic approaches, including medical treatment, covered stent angioplasty, coil insertion, and surgery have been described [9]. Checchia et al. [10] in 1997 described 10 pediatric patients who underwent cardiac transplantation for severe ischemic heart disease as a sequela of KD, showing that this technique is feasible and can benefit the small subgroup of patients who are not candidates for surgical or percutaneous revascularization or those with severe irreversible myocardial dysfunction. To the best of our knowledge, our clinical case is the first to describe heart transplantation as a treatment option for an adult patient with nonoperable CAAs refractory to conventional medical therapy.

### 3.1. Follow‐Up

The postoperative course was complicated by acute renal failure requiring hemodialysis, which was discontinued after 2 months following recovery of renal function. No further complications occurred and the patient was discharged 5 weeks after the transplant. To date, serial endomyocardial biopsies have never shown signs of cellular rejection, and echocardiographic evaluations document preserved biventricular function. The patient has lost weight during follow‐up and reports a significant improvement in exercise tolerance.

## 4. Conclusions

This case highlights a rare and complex presentation of giant CAAs in an adult patient, ultimately managed through orthotopic heart transplantation. The patient′s clinical course underscores the challenges in diagnosing and treating CAAs, especially when conventional surgical and percutaneous interventions are not feasible. Our multidisciplinary approach, utilizing advanced imaging techniques, was crucial for accurate diagnosis and therapeutic planning. The successful heart transplantation and subsequent favorable outcomes, including recovery from acute renal failure and significant improvement in exercise tolerance, demonstrate the potential viability of this treatment in similar cases. This case adds to the limited literature on heart transplantation as a therapeutic option for nonoperable CAAs.

**Nomenclature**
CAA: coronary artery aneurysm
CABG: coronary artery bypass grafting
cCTA: coronary computed tomographic angiography
CMR: cardiac Magnetic Resonance
NYHA: New York Health Association
TTE: transthoracic echocardiography

## Funding

Open access publishing was facilitated by the Aziende Socio Sanitarie Territoriale Papa Giovanni XXIII, as part of the Wiley–SBBL agreement.

## Consent

No written consent has been obtained from the patients as there is no patient identifiable data included in this case report/series.

## Conflicts of Interest

The authors declare no conflicts of interest.

## Data Availability

The authors declare that the data supporting the findings of this study are available within the paper.
